# *Pasteurella multocida* infection induces blood–brain barrier disruption by decreasing tight junctions and adherens junctions between neighbored brain microvascular endothelial cells

**DOI:** 10.1186/s13567-024-01351-5

**Published:** 2024-08-29

**Authors:** Lin Lin, Haixin Bi, Jie Yang, Yuyao Shang, Qingjie Lv, Dajun Zhang, Xi Huang, Mengfei Zhao, Fei Wang, Lin Hua, Huanchun Chen, Bin Wu, Xiangru Wang, Zhong Peng

**Affiliations:** 1 National Key Laboratory of Agricultural Microbiology, College of Veterinary Medicine, Huazhong Agricultural University, Wuhan, 430070 China; 2 Hubei Hongshan Laboratory, Wuhan, 430070 China; 3 Frontiers Science Center for Animal Breeding and Sustainable Production, The Cooperative Innovation Center for Sustainable Pig Production, Wuhan, 430070 China

**Keywords:** *Pasteurella multocida*, blood–brain barrier, disruption, paracellular migration, meningitis

## Abstract

Meningitis induced by *Pasteurella multocida* has been substantially described in clinical practice in both human and veterinary medicine, but the underlying mechanisms have not been previously reported. In this study, we investigated the influence of *P. multocida* infection on the permeability of the blood–brain barrier (BBB) using different models. Our in vivo tests in a mouse model and in vitro tests using human brain microvascular endothelial cell (hBMEC) model showed that *P. multocida* infection increased murine BBB permeability in mice and hBMEC monolayer permeability. Furthermore, we observed that *P. multocida* infection resulted in decreased expression of tight junctions (ZO1, claudin-5, occludin) and adherens junctions (E-cadherin) between neighboring hBMECs. Subsequent experiments revealed that *P. multocida* infection promoted the activation of hypoxia inducible factor-1α (HIF-1α)/vascular endothelial growth factor A (VEGFA) signaling and NF-κB signaling, and suppressed the HIF-1α/VEGFA significantly remitted the decrease in ZO1/E-cadherin induced by *P. multocida* infection (*P* < 0.001). NF-κB signaling was found to contribute to the production of chemokines such as TNF-1α, IL-β, and IL-6. Additionally, transmission electron microscopy revealed that paracellular migration might be the strategy employed by *P. multocida* to cross the BBB. This study provides the first evidence of the migration strategy used by *P. multocida* to traverse the mammalian BBB. The data presented herein will contribute to a better understanding of the pathogenesis of the zoonotic pathogen *P. multocida*.

**Supplementary Information:**

The online version contains supplementary material available at 10.1186/s13567-024-01351-5.

## Introduction

Bacterial meningitis is characterized by inflammation of the meninges, caused by various bacterial pathogens [1]. This disease is a significant global health concern, with a mortality rate of up to 54% in low-income countries [2]. *Streptococcus pneumoniae*, *Neisseria meningitidis*, and *Haemophilus influenzae* type b are the primary bacterial species responsible for bacterial meningitis worldwide [3]. The ability of bacteria to breach the blood–brain-barrier (BBB) is a crucial step in the development of the disease [3, 4]. Serving as a vital physiological barrier in mammals, the BBB plays a critical role in safeguarding the central nervous system (CNS) against toxins, pathogens, inflammation, injury, and disease [5]. The BBB primarily consists of brain microvascular endothelial cells (BMECs) that are interconnected by tight junctions (e.g., ZO1), adherens junctions (e.g., E-cadherin), and associated proteins [4]. Bacterial pathogens employ three main strategies that cross the BBB following their interaction with BMECs: the transcellular pathway (direct invasion and traversal through BMECs), paracellular pathway (disruption of intercellular junctions and/or induction of cell damage), or the Trojan-horse mechanism (intracellular transport within infected phagocytes) [6].

*Pasteurella multocida*, a pathogen phylogenetically related to *H. influenzae*, is an important zoonotic pathogen that causes various diseases in animals and humans [7]. Clinical manifestations associated with *P. multocida* infection can be broadly categorized into two types: (1) respiratory disorders, such as atrophic rhinitis in pigs and rabbits and pneumonia in a various animal species; (2) bloodstream infections, including hemorrhagic septicemia in cattle and other ruminants, as well as fowl cholera in poultry and wild birds [8]. A substantial number of case reports have documented neurological signs induced by this versatile bacterial pathogen in both human and veterinary medicine [9–13], suggesting the potential of *P. multocida* to cross the BBB and cause CNS infection. However, laboratory studies investigating this hypothesis and elucidating the underlying mechanisms are lacking. In this study, we aimed to investigate whether *P. multocida* could breach the BBB and explore  the mechanisms involved, using mouse and human BMEC (hBMEC) models. Our results demonstrate that *P. multocida* infection disrupts the BBB by invading brain microvascular endothelial cells.

## Materials and methods

### Bacterial strains, cell lines and culture conditions

*Pasteurella multocida* strains used in this study include strain HuN001 (GenBank accession no. CP073238) and C09. Strain HuN001 was isolated from the sputum of a patient with pneumonia [14], while strain C09 was isolated from the pharyngeal swab of a cat with respiratory symptoms. Both strains are capsular type A and do not produce *Pasteurella multocida* toxin (PMT), a dermonecrotic toxin. Unless specified otherwise, *P. multocida* was cultured on tryptic soy agar (TSA; Becton, Dickinson and Company, MD, USA) or in tryptic soy broth (TSB; Becton, Dickinson and Company, MD, USA) supplemented with 5% newborn bovine serum (Tianhang, Hangzhou, China) at 37 °C for a least 12 h. Human brain microvascular endothelial cells (hBMECs) were maintained in RPMI 1640 medium (Gibco, ThermoFisher, Waltham, MA, USA) supplemented with 10% fetal bovine serum (Gibco, USA) in 5% CO_2_ atmosphere at 37 °C.

### Mouse experiment and ethics statements

**Figure 1:**
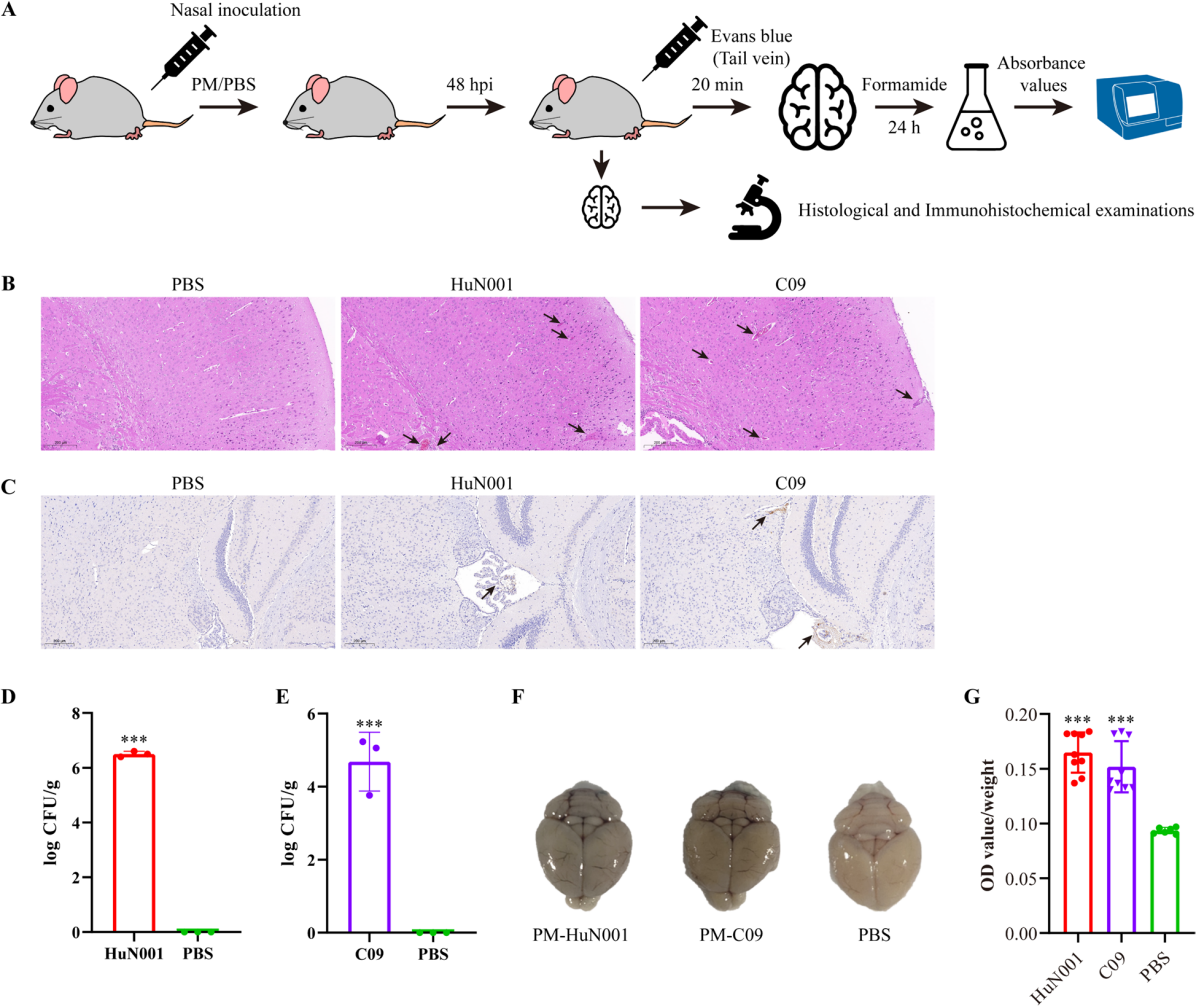
**In vivo tests in mouse models assessing the influence of *Pasteurella multocida* infection on the blood–brain barrier. A** Study design of the mouse experiments. Mice were inoculated with different *P. multocida* strains or PBS. Evans blue (EB) was injected at 48 h post inoculation, and after 20 min, the mice were euthanized. Murine brains were collected to quantify EB dyes by measuring the absorbance values of optical density at 620 nm [OD_620_]. **B** Pathological damages (marked with black arrows) in the brains of mice inoculated with different *P. multocida* strains or PBS, as characterized by histological examinations (bar = 200 μm). **C** Expression of von Willebrand factor (vWF) (indicated by black arrows) in the brains of *P. multocida*-infected mice (HuN001, C09) and PBS treated mice, as characterized by immunohistochemical examinations (bar = 200 μm). **D** Recovery of *P. multocida* HuN001 from the brains of bacterium-infected mice (HuN001) and PBS treated mice. **E** Recovery of *P. multocida* C09 from the brains of bacterium-infected mice (C09) and PBS treated mice. **F** Visualization of the brains obtained from mice inoculated with *P. multocida* strains (HuN001 and C09), or the control (PBS), showing brain staining with EB dye. **G** Quantification of EB in the brain obtained from *P. multocida*-infected mice and control mice. PM refers to *P. multocida*.

To investigate the potential of *P. multocida* to induce BBB disruption in vivo, mouse experiments were conducted at the Laboratory Animal Center at Huazhong Agricultural University (Wuhan, China). The study received approval from the University Ethics Committees (approval no. HZAUMO-2023–0235). The experimental design involved 5–6-week-old female mice divided into three groups: G1, G2, and G3, each consisting of eight mice. In G1, mice were intranasally inoculated with *P. multocida* HuN001 at a dose of 50 colony-forming units (CFU) per mouse. In G2, mice were intranasally inoculated with *P. multocida* strain C09 at a dose of 5 × 10^7^ CFU per mouse. As a control, mice in G3 received an intranasal administration of PBS at a volume of 50 µL per mouse (Figure 1A). The doses for challenge were determined based on the lethal doses of these two strains tested on mice in our laboratory previously. At 48 h post-inoculation (hpi), three mice from each group were euthanized, and their brain tissues were collected for histological examination and bacterial recovery. Immunohistochemical examinations were also conducted on brain tissues using a von Willebrand factor (vWF) antibody (1:100) (Abcam, UK), following previously described methods [15]. Another five mice from each group received an injection of Evans Blue dye (Sigma, USA) at a dose of 30 mg/kg body weight through the tail vein routine. After 40 min, all mice were euthanized, and the dye in the brains was extracted using formamide (2 mL) at 55 °C for 24 h (Figure 1A). The changes in BBB permeability were assessed by measuring the absorbance values (optical density at 620 nm [OD_620_]) of the extracted solutions [16].

### Dextran-based trans-well permeability assay

To evaluate the impact of *P. multocida* infection on the barrier function of hBMECs, a dextran-based transwell permeability assay was conducted following a previously described protocol [15]. Briefly, approximately 1 × 10^5^ hBMECs in 200 µL of antibiotic-free RPMI 1640 medium were seeded onto 24-well cell culture inserts (Labselect, Hefei, China). The cells were cultured for 36 h in a 5% CO_2_ atmosphere at 37 °C. Subsequently, the cells were incubated with 200 µL of antibiotic-free RPMI 1640 containing *P. multocida* (HuN001 or C09) at a multiplicity of infection (MOI) of 200. Additionally, 10 μM of 70-kDa fluorescein isothiocyanate (FITC; Sigma, St. Louis, MO, USA) was added to the medium. The cells were then incubated at 37 °C for 12 h under 5% CO_2_ atmosphere. After incubation, 100 µL of the medium from the basal chamber was transferred to a black well plate (Greiner Bio-One, Germany). The permeability of dextran was determined based on the results obtained from the plate in a Victor Nivo multimode plate reader (PerkinElmer, Waltham, MA, USA), measuring the fluorescence intensity at excitation/emission wavelengths of 490 nm/520 nm.

### Quantitative real-time PCR

HBMEC monolayers were infected with *P. multocida* HuN001 (200 MOI) or C09 (200 MOI) and incubated at 37 °C under 5% CO_2_ for 4 h (for NF-κB) or 12 h. As a control, cells treated with PBS under the same conditions were included. After washing three times with PBS, total RNAs was extracted using the TRIzol reagent protocol (Invitrogen, Thermo Fisher, Waltham, MA, USA). Subsequently, cDNAs was synthesized using a PrimeScript RT reagent kit with gDNA Eraser (TAKARA, Japan), and the synthesized cDNAs was used as a template for quantitative real-time PCR (qPCR) assays to detect the transcriptional levels of various genes, including NF-κB, hypoxia inducible factor-1α (HIF-1α), vascular endothelial growth factor (VEGFA), tight junctions (ZO1, claudin-5, occludin), adherens junctions (E-cadherin), and chemokines (IL-1β, IL-6, TNF-α). Glyceraldehyde-3-phosphate dehydrogenase (GAPDH) was used as a reference gene. Primers used for qPCR are listed in Additional file 1. All experiments were repeated thrice.

### Western-blotting

To examine the expression of proteins, hBMEC monolayers were inoculated with *P. multocida* HuN001 (200 MOI), C09 (200 MOI), or PBS and incubated at 37 °C under 5% CO_2_ for 4 h (for phosphorylated P65, p-P65) or 12 h. Cells were then lysed using radioimmunoprecipitation assay (RIPA) buffer (Beyotime, China) containing protease inhibitors. The lysates were centrifuged at 4 °C, 12 000 rpm for 10 min. The protein concentration in the harvested lysates was quantified using a commercial BCA Protein Assay Kit (Beyotime, China). The proteins were separated on 10% or 12.5% sodium dodecyl sulfate–polyacrylamide gel electrophoresis (SDS-PAGE) gels and transferred onto polyvinylidene difluoride (PVDF) membranes (Bio-Rad, USA). The membranes were washed with Tris-buffered saline with Tween 20 (TBST) for five times then blocked with 5% BSA in TBST for 3 h at room temperature. Subsequently, the membranes were incubated overnight at 4 °C with specific antibodies, including HIF-1α antibody (1:1000) (catalog no. NB100-134; Novus Biologicals, USA), VEGFA polyclonal antibody (1:5000) (catalog no. 19003–1-AP; Proteintech, China), ZO1 polyclonal antibody (1:5000) (catalog no. 21773–1-AP; Proteintech, China), E-cadherin monoclonal antibody (1:1000) (catalog no. P12830; Cell Signaling, USA), Phospho-NF-κB P65 (1:1000) (catalog no. #3033; Cell Signaling, USA), and GAPDH monoclonal antibody (1:20,000) (catalog no. 60004–1-lg; Proteintech, China). Following another round of TBST washing, the membranes were incubated with species-specific horseradish peroxidase-conjugated antibodies for 1 h at room temperature. Finally, the blots were visualized using enhanced chemiluminescence (ECL) reagents (Beyotime, China), and the bands were quantified using ImageJ software (v1.8.0). The results were analyzed as the relative immunoreactivity of each protein, normalized to the respective loading controls. Additional requirements for the examination of HIF-1α have been previously described [15].

### siRNA transfection and bacterial infection

To investigate the influence of HIF-1α and NF-κB on barrier function changes in hBMECs induced by *P. multocida*, specific small interfering RNAs (siRNAs) against HIF-1α or NF-κB (Additional file 1) were synthesized. These siRNAs were transfected into hBMECs using Lipofectamine 2000 reagent (Invitrogen, USA) following the manufacturer’s instructions. A scrambled RNA sequence at the same concentration (100 nM) was transfected as a control. The efficacy of the siRNAs in suppressing the expression of the target genes was examined using qPCR. Monolayers of both siRNA-transfected cells and control cells were then infected with *P. multocida* HuN001 and C09 at a MOI of 200 for 12 h. The transcriptional levels of different genes, including ZO1, E-cadherin, HIF-1α, NF-κB, TNF-1α, IL-β, and IL-6, were detected using qPCR. Additionally, the expression of ZO1 and E-cadherin was determined using western blotting, as described above.

### Immunofluorescence

To observe and compare the expression of ZO1 in bacteria-infected cells and PBS-treated cells, monolayers of hBMECs were inoculated with *P. multocida* HuN001 (MOI = 200), C09 (MOI = 200), or PBS (50 µL) for 12 h at 37 °C under 5% CO_2_. After washing with precooled PBS to remove free bacteria, the cells were fixed with precooled formaldehyde for 2 h and blocked in 5% BSA at room temperature for 2 h. Subsequently, the cells were incubated overnight at 4 °C with ZO1 polyclonal antibody (1:2000). After washing with precooled PBS, cells were incubated with CoraLite488-conjugated Goat Anti-Rabbit IgG(H + L) (catalog no. SA00013-2; Proteintech, China) at 37 °C for 30 min in dark place. Finally, the cells were incubated with antifade mounting medium containing 4′,6-diamidino-2-phenylindole (DAPI) (Beyotime, China) for 30 min at room temperature in the dark place. The expression of ZO1 was observed under an inverted fluorescence microscope (Olympus BX53, Japan).

### Laser scanning confocal microscope

Laser scanning confocal microscopy was used to examine the influence of *P. multocida* infection on the phosphorylation of NF-κB P65 in hBMECs. To achieve this, Monolayers of hBMECs were incubated with *P. multocida* HuN001 (MOI of 200), C09 (MOI of 200), or PBS (50 µL) at 37 °C for 4 h, followed by washing with precooled PBS to remove free bacteria. The cells were then fixed in pre-cooled formaldehyde for 2 h and blocked with 5% BSA for 1 h at room temperature. After washing, the cells were incubated overnight at 4 °C with a Phospho-NF-κB P65 antibody (1:2000) (catalog no. #3033; Cell Signaling Technology). Following washing with precooled PBS, the cells were incubated with CoraLite488-conjugated Goat Anti-Rabbit IgG(H + L) at 37 °C for 1 h in a dark place. Finally, the cells were incubated with antifade mounting medium containing 4′,6-diamidino-2-phenylindole (DAPI) (Beyotime, China) for 30 min at room temperature in the dark place. The phosphorylation of NF-κB P65 was observed under a Zeiss LSM 800 Confocal Laser Scanning Microscope and was analyzed using NIS-Elements Viewer 4.20 (Nikon, Tokyo, Japan).

### Transmission electron microscope

To examine the strategy used by *P. multocida* to migrate the barrier formed by hBMECs, samples were prepared for transmission electron microscopy following the method described in a previously published article [17]. Briefly, monolayers of hBMECs were incubated with *P. multocida* HuN001 (MOI = 200) or PBS (50 µL) at 37 °C for 3 h. After washing three times with PBS, the bacterium-infected cells or PBS-treated cells were divided into two groups. One group of cells was fixed using a commercial electron microscope fixative (code: G1102, Servicebio, Wuhan, China) for 5 min in dark place, while another group of cells was treated with kanamycin (100 μg/mL) and ampicillin (100 μg/mL) for 30 min at 4 °C to remove extracellular bacteria. After washing thrice with PBS, antibiotic-treated cells were fixed using a commercial electron microscope fixative for 5 min in the dark. Thereafter, the cells were scraped and collected by centrifugation at 2000 rpm for 5 min. The cells were then resuspended in fresh electron microscope fixative and fixed for 30 min in the dark. The fixed cells were then sent to the National Key Laboratory of Agricultural Microbiology Core Facility at Huazhong Agricultural University for slide preparation. The prepared slides were observed under a 100-kV transmission electron microscope (H-7650, HITACHI, Japan).

### Statistical analyses

Statistical analysis was performed using the *multiple-t-test* strategy in GraphPad Prism 8.0 (GraphPad Software, San Diego, CA, USA). Data represent mean ± standard deviation (SD). The significance level was set at a *P* value of < 0.05 (*), a *P* value of < 0.01 (**), or a *P* value of < 0.001 (***).

## Results

### *Pasteurella multocida* infection enhances the integrity of blood–brain barrier in mouse and cell models

To investigate whether *P. multocida* could disrupt BBB integrity, SPF mice were intranasally challenged with *P. multocida* strains HuN001 or C09 (Figure 1A). Subsequently, we examined brain damages conducted *P. multocida* recovery experiments. Histological examination revealed thickened blood vessel walls, increased inflammatory cell infiltration, proliferated vasculature, and hemorrhage in the brains of *P. multocida* infected mice compared to PBS-treated mice (Figure 1B). Immunohistochemical examinations using the von Willebrand factor (vWF) antibody also demonstrated increased expression of vWF in the brains of *P. multocida* infected mice compared to that in PBS-treated mice (Figure 1C). As expected, purified *P. multocida* isolates were recovered from the brains of bacteria-challenged mice, while no bacterial colonies were recovered from the brains of PBS-treated mice (Figure 1D and E). Assessment of the integrity of the murine BBB using Evans blue dye showed a significantly higher concentration of Evans blue in the brains of bacteria-challenged mice compared to PBS-treated mice (Figure 1F and G). Collectively, these findings revealed an increased permeability of the murine BBB during *P. multocida* infection.

**Figure 2:**
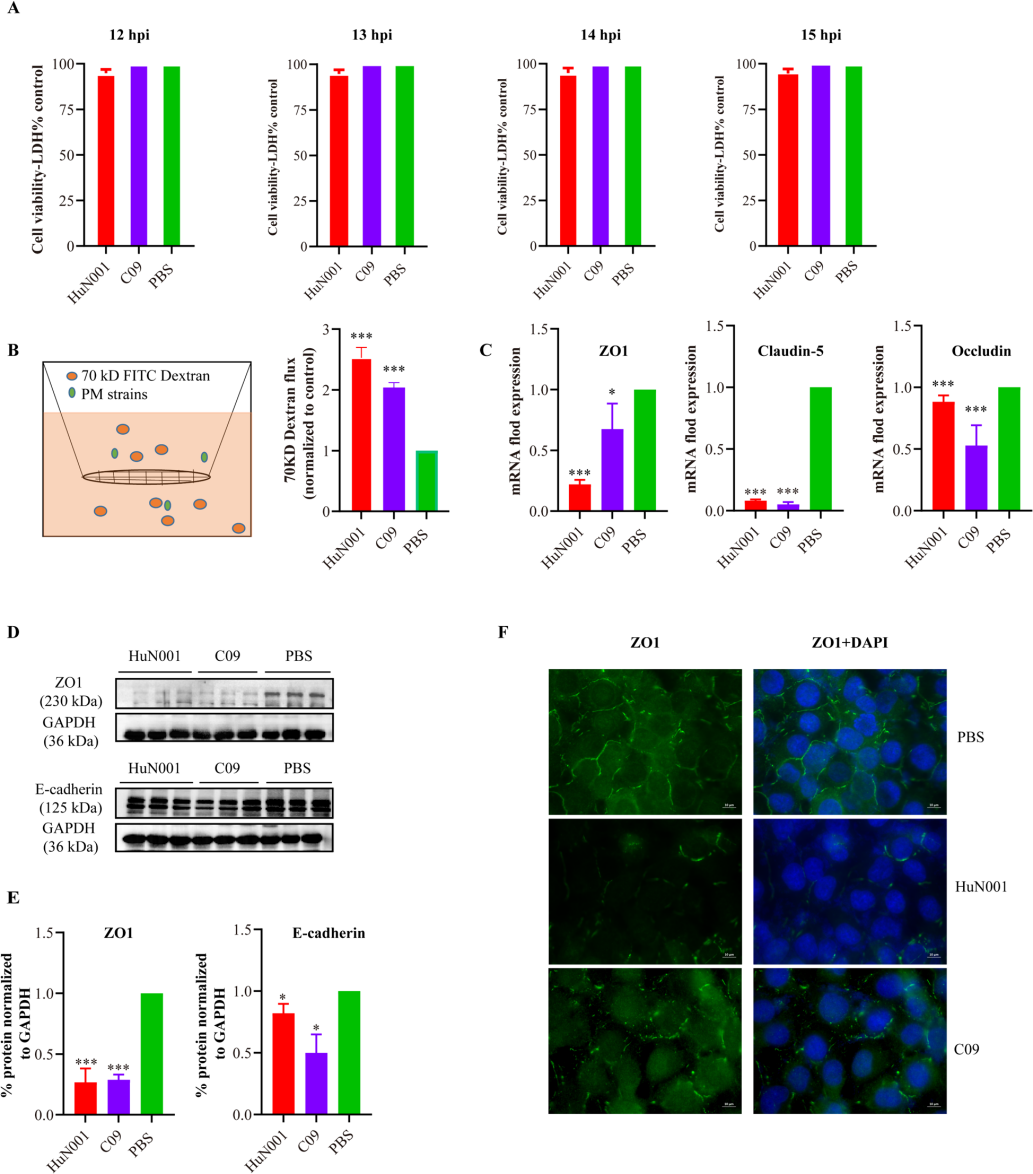
**In vitro tests in human brain microvascular endothelial cell (hBMEC) models assessing the influence of** ***Pasteurella multocida*** **infection on hBMEC monolayers. A** In vitro cytotoxicity assays revealing the conditions of hBMECs at 12-, 13-, 14-, and 15-h post *P. multocida*-infection. **B** Dextran-based transwell permeability assay showing the increase in the vascular permeability of hBMECs induced by *P. multocida* (PM) infection. Cells inoculated with PBS were included as control for the assay. **C** Transcription of tight junctions (ZO1, claudin-5, occludin) in *P. multocida* infected cells compared to PBS-treated cells, as determined by qPCR. **D** Western blots demonstrating the expression of ZO1 and E-cadherin in *P. multocida* infected cells and PBS-treated cells; **E** Quantification of western blots demonstrating the expression of ZO1 and E-cadherin in *P. multocida* infected cells and PBS-treated cells using ImageJ software. **F** Immunofluorescence images showing the reduced expression of ZO-1 in hBMECs infected with *P. multocida* strains compared to control PBS-treated cells. DAPI (49,6-diamidino-2-phenylindole) was used for nuclear staining.

Next, we confirmed the in vivo findings using hBMEC models. In vitro cytotoxicity assays showed that hBMEC monolayers remained in good condition at 12, 13, 14, and 15-h post bacteria-inoculation (Figure 2A). Dextran-based transwell permeability assays demonstrated a significant increase in the permeability of hBMEC monolayers following inoculation with *P. multocida* HuN001 or C09 (Figure 2B). Immunofluorescence staining of tight junctions (ZO1, claudin-5, occludin) and adherens junctions (E-cadherin) between neighboring hBMECs also revealed a significant decrease in the expression of these molecules in bacteria-infected cells compared to PBS-treated cells (Figure 2C–F). These findings suggest that *P. multocida* infection induces BBB disruption.

### HIF-1α/VEGFA signaling is involved in the disruption of hBMEC monolayer permeability induced by *Pasteurella multocida* infection

**Figure 3:**
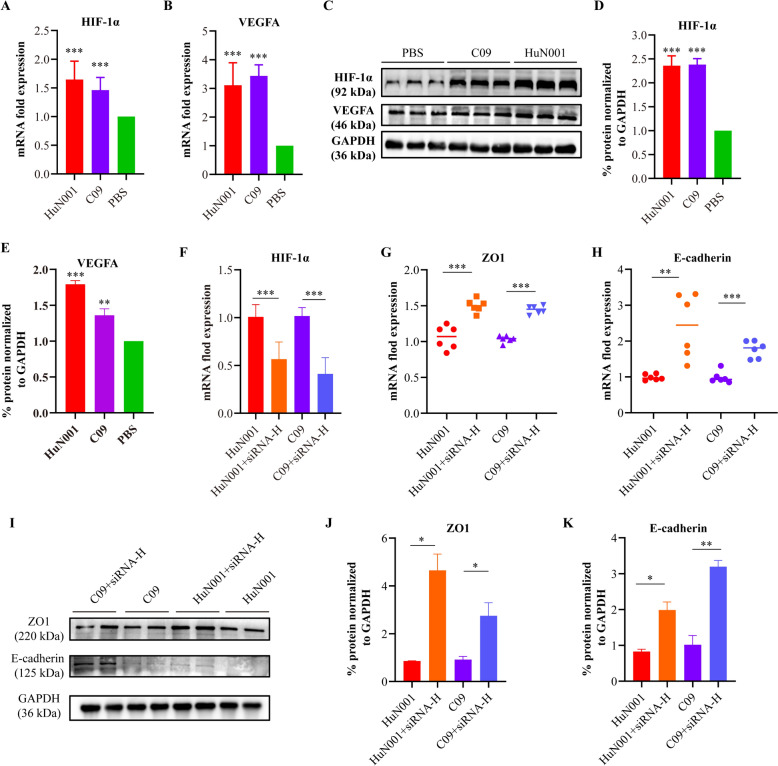
**Activation of the hypoxia inducible factor-1α (HIF-1α)/vascular endothelial growth factor A (VEGFA) signaling in human brain microvascular endothelial cells (hBMECs) induced by** ***Pasteurella multocida*** **infection. A** Transcription of HIF-1α in *P. multocida* infected cells compared to PBS-treated cells, as determined by qPCR. **B** Transcription of VEGFA in *P. multocida* infected cells compared to PBS-treated cells, as determined by qPCR. **C** Western blots demonstrating the expression of HIF-1α and VEGFA in *P. multocida* infected cells and PBS-treated cells. **D** Quantification of western blots demonstrating the expression of HIF-1α in *P. multocida* infected cells and PBS-treated cells by ImageJ software. **E** Quantification of western blots demonstrating the expression of VEGFA in *P. multocida* infected cells and PBS-treated cells by ImageJ software. **F** Efficacy of specific siRNAs in suppressing the expression of HIF-1α in hBMECs post *P. multocida* infection; siRNA-H refers to siRNAs in suppressing the expression of HIF-1α in hBMECs. **G** Transcription of ZO1 in HIF-1α-knockdown cells compared to the wild type cells post *P. multocida* infection. **H** Transcription of E-cadherin in HIF-1α-knockdown cells compared to the wild type cells post *P. multocida* infection. **I** Western blots demonstrating the expression of ZO1 and E-cadherin in HIF-1α-knockdown cells compared to the wild type cells post *P. multocida* infection. **J**, **K** Quantification of western blots demonstrating the expression of ZO1 (**J**) and E-cadherin (**K**) in HIF-1α-knockdown cells compared to the wild type cells post *P. multocida* infection.

Many studies have reported the involvement of HIF-1α/VEGFA signaling in tissue barrier disruption caused by bacterial pathogens [4, 15, 18, 19]. Therefore, we examined the status of this signaling pathway in hBMECs after *P. multocida* infection. The results demonstrated a significant increase in the transcription and expression of both HIF-1α and VEGFA in bacteria-inoculated cells compared to PBS-treated cells (*P* < 0.001, Figure 3A–E), indicating the activation of HIF-1α/VEGFA signaling. To determine whether the activation of HIF-1α/VEGFA signaling is associated with the disruption of hBMEC monolayer permeability induced by *P. multocida* infection, we used specific small interfering RNAs (siRNAs) to knock down the expression of HIF-1α and detected the expression of tight junctions and/or adherens junctions between neighboring cells after bacteria inoculation (Figure 3F). The results revealed that HIF-1α knockdown significantly restored the decrease in ZO1/E-cadherin induced by *P. multocida* infection (Figure 3G–K).

### NF-κB signaling contributes to the production of chemokines in hBMEC monolayers induced by *Pasteurella multocida* infection

**Figure 4:**
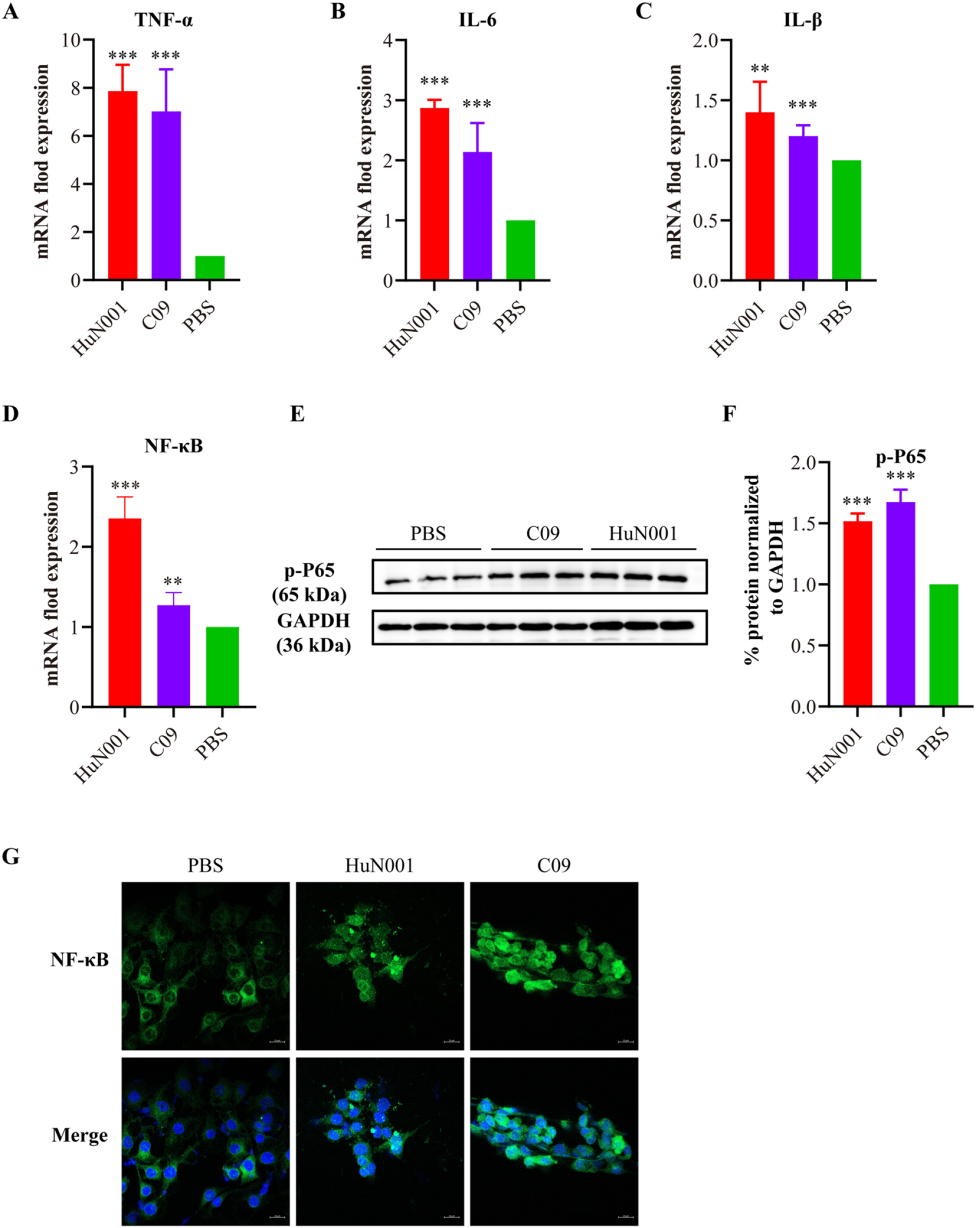
***Pasteurella multocida *infection induces the activation of NF-κB signaling in human brain microvascular endothelial cells (hBMECs). A–C** Transcription of TNF-α (**A**), IL-6 (**B**), and IL-1β (**C**) in *P. multocida* infected cells compared to PBS-treated cells, as determined by qPCR. **D** Transcription of NF-κB in *P. multocida* infected cells compared to PBS-treated cells, as determined by qPCR. **E** Western blots demonstrating the expression of phosphorylated NF-κB P65 (p-P65) in *P. multocida* infected cells and PBS-treated cells. **F** Quantification of western blots demonstrating the expression of p-P65 in *P. multocida* infected cells and PBS-treated cells by ImageJ software. **G** Confocal laser scanning microscopy images showing the movement of NF-κB P65 into the cell nucleus in *P. multocida* infected cells and PBS-treated cells.

**Figure 5:**
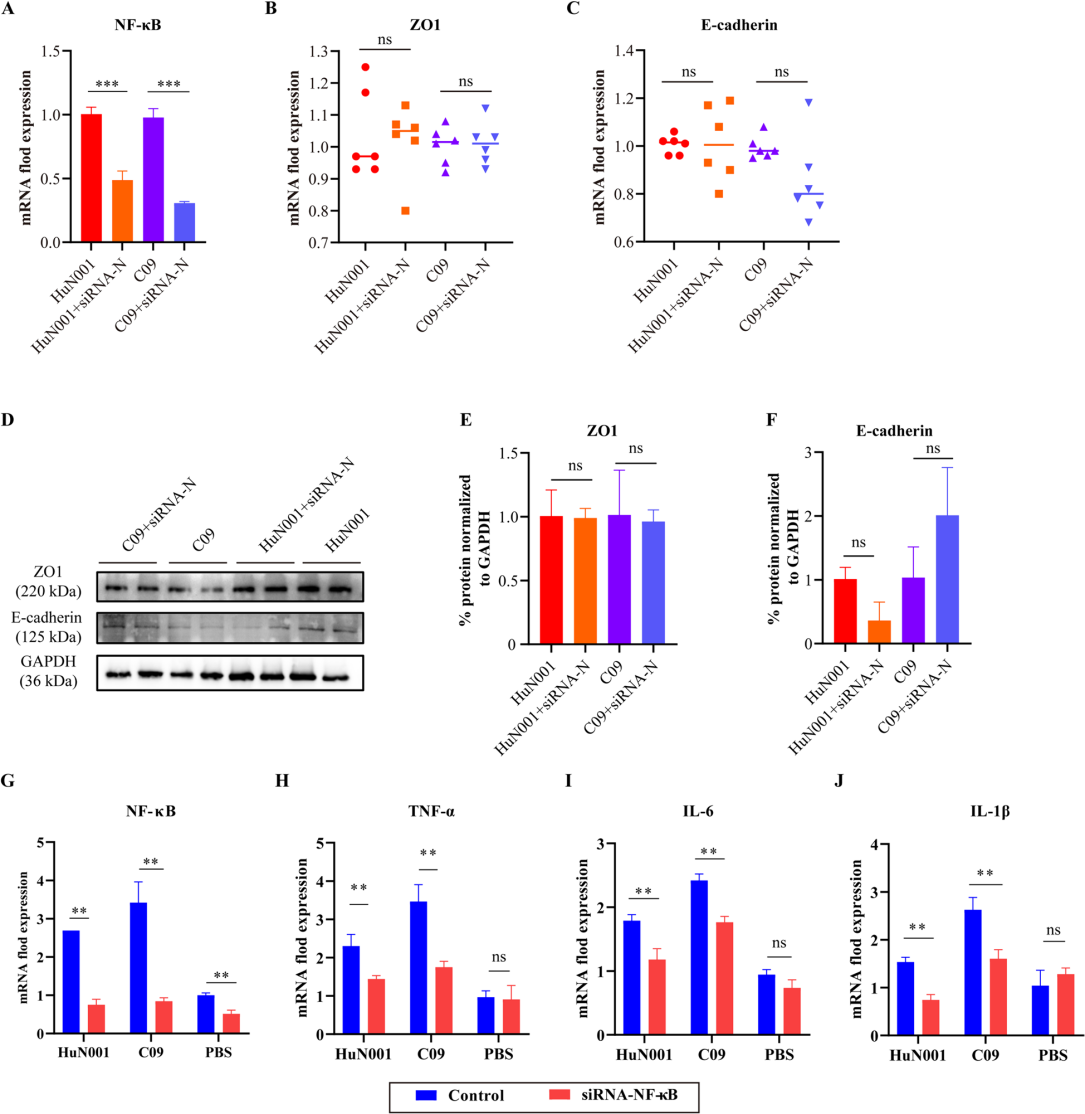
**Influence of the NF-κB signaling on the expression of ZO1, E-cadherin, and cytokines in human brain microvascular endothelial cells (hBMECs) induced by *Pasteurella multocida*. A**, **G** Efficacy of specific siRNAs in suppressing the expression of NF-κB in hBMECs post *P. multocida* infection; siRNA-N refers to siRNAs in suppressing the expression of NF-κB in hBMECs. **B** Transcription of ZO1 in NF-κB-knockdown cells compared to the wild type cells post *P. multocida* infection. **C** Transcription of E-cadherin in NF-κB-knockdown cells compared to the wild type cells post *P. multocida* infection. **D** Western blots demonstrating the expression of ZO1 and E-cadherin in NF-κB-knockdown cells compared to the wild type cells post *P. multocida* infection; **E**, **F** Quantification of western blots demonstrating the expression of ZO1 (**E**) and E-cadherin (**F**) in NF-κB-knockdown cells compared to the wild type cells post *P. multocida* infection; **H**–**J** Transcription of TNF-α (**H**), IL-6 (**I**), and IL-1β (**J**) in NF-κB-knockdown cells compared to the wild type cells post *P. multocida* infection.

To examine the inflammatory reactions in hBMECs during *P. multocida* infection, we detected the production of common chemokines such as TNF-1α, IL-β, and IL-6. The results revealed significantly higher transcriptional levels of TNF-1α, IL-β, and IL-6 in bacteria-inoculated cells compared to PBS-treated cells (*P* < 0.001, Figure 4A–C). Activation of NF-κB signaling was also observed, as evidenced by increased transcription of NF-κB and phosphorylation of p65 (p-p65) in bacteria-inoculated cells compared to PBS-treated cells (Figure 4D–G). Subsequently, we used specific siRNAs to knock down NF-κB activity and detected the production of chemokines as well as the expression of tight junctions and/or adherens junctions after *P. multocida* infection. The results demonstrated that NF-κB knockdown led to a decrease in chemokine-transcription (TNF-1α, IL-β, and IL-6) in hBMECs during *P. multocida* infection but had no impact on the expression of ZO1/E-cadherin (Figure 5).

### *Pasteurella multocida* crosses the blood–brain barrier through the paracellular routine

**Figure 6:**
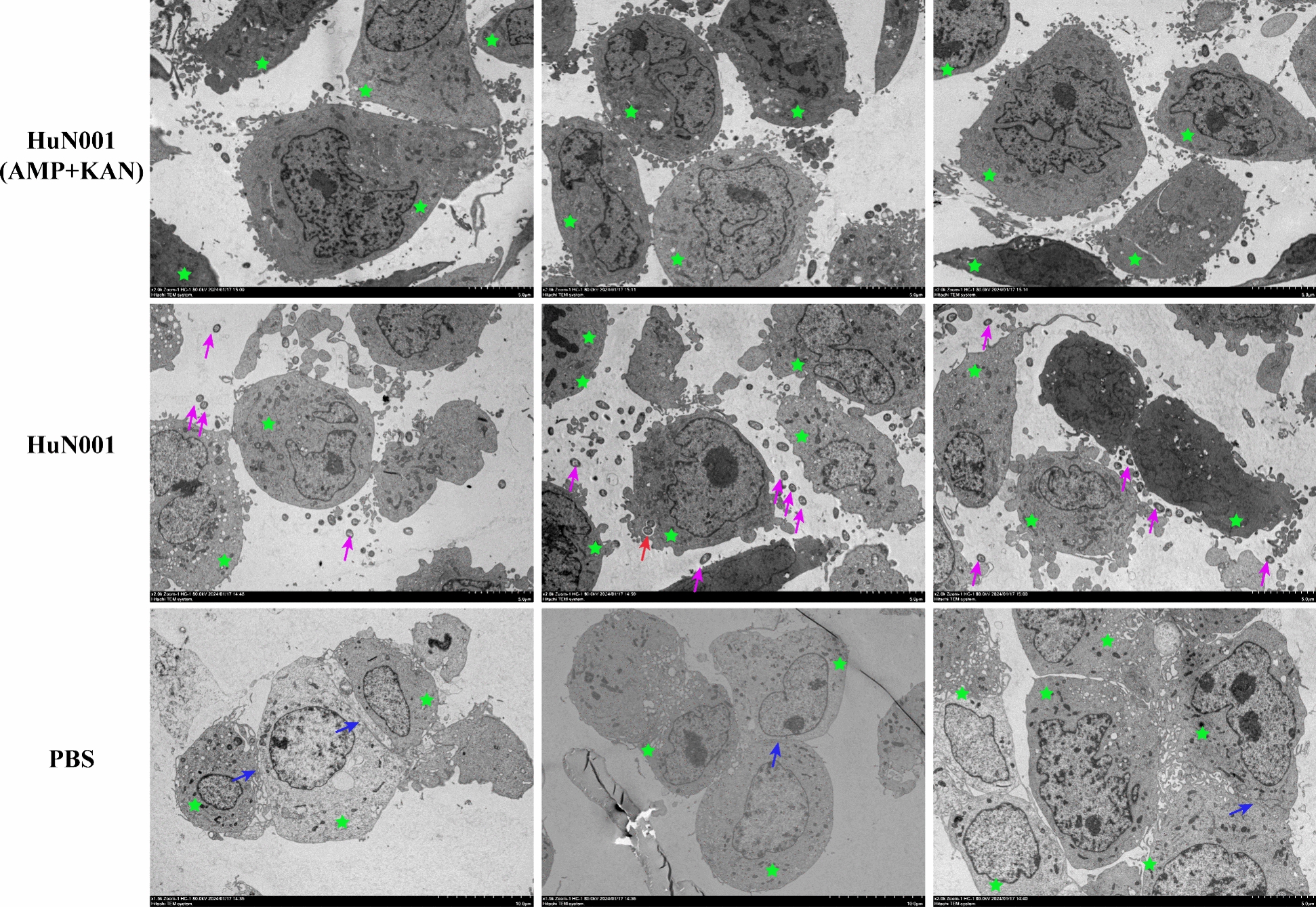
**Transmission electron microscope examination of the strategy of *Pasteurella multocida* used for migrating the barrier formed by human brain microvascular endothelial cells (hBMECs).** Cells are shown with green stars, bacterial strains in the area between neighbored hBMECs are showed with purple arrows, while those invading the cells are shown with red arrows; blue arrows show tight junctions and adherens junctions between neighbored hBMECs; bars = 5 µm; AMP: ampicillin, KAN: kanamycin.

To understand the strategy by which *P. multocida* crosses the BBB, transmission electron microscopy was used to observe bacterial migration in hBMECs following a previously described protocol [17]. The results revealed that inoculation with *P. multocida* caused the disruption of tight junctions and adherens junctions between neighboring hBEMCs, and most of the inoculated bacterial strains were observed within the intercellular space (Figure 6). Conversely, there were almost no bacterial strains detected within the hBEMCs (Figure 6). These findings suggest that *P. multocida* may use a paracellular pathway strategy to migrate through the BBB.

## Discussion

In this study, we provide the first evidence that *P. multocida* is capable of invading hBMECs and partially elucidates the related mechanisms in cell models. The recognition of *P. multocida* as a zoonotic pathogen dates back to 1930 [20]. The impact of this Gram-negative bacterium on human health has often been overlooked due its low fatality rate and ability to easily treat most cases of human pasteurellosis [7]. However, considering the escalating prevalence of companion animals like cats and dogs, as well as food-producing animals such as pigs and cattle, due to global economic and social development, public health concerns regarding *P. multocida* should not be ignored [7, 21]. Animal exposure remains one of the primary causes of human pasteurellosis [7]. While various symptoms have been associated with *P. multocida* infections in humans, neurological signs or meningitis are commonly observed in clinical diagnosis [10–12]. This study investigated the role of *P. multocida* infection in disrupting the mammalian BBB and partially uncovered the underlying mechanisms. This study is believed to be the first to demonstrate the migration strategy of *P. multocida* across the mammalian BBB.

In this study, a mouse model was employed to assess the disruption of the BBB induced by *P. multocida*. The use of mice as an in vivo model is not only due to their widespread utilization in studies on meningitis caused by other bacterial species [17, 22], but also because rodents are natural hosts of *P. multocida*, and recent reports have documented infections in the brains of wild rodents caused by *P. multocida* [23]. Our findings demonstrated that the inoculation of *P. multocida* strains from different hosts resulted in pathological damage in the brains of mice after the challenge, and bacterial strains were recovered from the brains of the infected animals, indicating that respiratory inoculation of *P. multocida* can cause brain injury to the brain and ultimately invade the animal’s brains. To provide further evidence, we injected Evans blue dye to evaluate the increase in the permeability of the murine BBB, as recommended by other studies [17, 22]. Evans blue, as an azo dye with a high affinity for serum albumin, is commonly used to assess changes in the permeability of physiological barriers [15]. The quantitative evaluation of BBB permeability can be achieved by measuring the fluorescence intensity of Evans blue in brain structures [22]. In our in vivo tests using mouse models, we observed a significantly higher density of Evans blue in the brains of mice challenged with *P. multocida* strains compared to those inoculated with PBS, indicating that *P. multocida* infections induce increased BBB permeability in the challenged mice. In addition to the human isolate HuN001, we also used a strain (C09) of *P. multocida* originating from cats in the present study. This decision was motivated by the fact that cases of human pasteurellosis, including meningitis, are frequently associated with *P. multocida* strains from pets, particularly cats [7, 12, 24]. Notably, the human isolate HuN001 was likely derived from a golden retriever dog owned by the patient, as indicated by admission records [14]. Although a small proportion of *P. multocida* strains produce the dermonecrotic toxin known as *Pasteurella multocida* toxin (PMT), these strains are primarily associated with progressive atrophic rhinitis in pigs and are rarely isolated in other cases of pasteurellosis [7, 8]. Therefore, we did not investigate toxigenic strains of *P. multocida* in this study.

BMECs, as the primary constituent of the BBB [5], have been widely used as an in vitro model to investigate the mechanisms by which pathogens disrupt or traverse the BBB [25–27]. In our study, using hBMECs as a model, we conducted dextran-based trans-well permeability assays, which revealed that *P. multocida* infection induced an increase in hBMEC monolayer permeability. This was evidenced by a higher presence of FITC-dextran in the bottom medium of the wells where *P. multocida* was inoculated, compared to the wells where the cells were treated with PBS. In addition, our examination demonstrated a reduction of in the transcription and/or expression of tight junctions (ZO1, claudin-5, and occludin) and adherens junctions (E-cadherin) in *P. multocida* infected hBMECs compared to PBS-treated cells. Tight junctions and adherens junctions between adjcent epithelial cells are known to play a critical role in maintaining the physiological function of tissue barriers, including the BBB [28, 29]. A decrease in the expression of these molecules signifies an increase in the permeability of tissue barriers [15, 25]. Consequently, the downregulation of tight junctions and adherens junctions might represent an important mechanism by which *P. multocida* crosses the BBB.

In this study, we also provided evidence that HIF-1α/VEGFA signaling is involved in the BBB dysfunction induced by *P. multocida*. This finding is consistent with previous studies investigating the mechanisms of meningitis induced by other bacterial species, such as *Streptococcus pneumoniae* [18]. Notably, the HIF-1α/VEGFA signaling pathway has also been reported to contribute to the disruption of other tissue barriers, including the respiratory and gut barriers, induced by bacterial pathogens [15, 30]. Additionally, the NF-κB signaling has also been implicated in BBB dysfunction [31, 32]. Consistently, we demonstrated that *P. multocida* infection significantly upregulated NF-κB signaling in hBMECs, leading to the production of chemokines such as TNF-1α, IL-β, and IL-6. Activation of the NF-κB signaling is recognized as one of the three hallmark features of bacterial meningitis [31]. Our current study also suggests that paracellular migration might be the strategy employed by *P. multocida* to cross the BBB. This is supported by transmission electron microscopy observation, in vitro cytotoxicity assays, and the examination of tight junctions and adherens junctions. In the paracellular strategy, bacterial pathogens disrupt intercellular junctions and migrate between neighboring cells [6].

In conclusion, the present study unveiled that respiratory tract infection with *P. multocida* leads to an elevation in BBB permeability and, for the first time, demonstrated the utilization of a paracellular migration strategy by *P. multocida* to traverse the mammalian BBB. These findings provide valuable insights into the pathogenesis of *P. multocida*, an emerging zoonotic pathogen with potential public health concerns that should not be ignored. It is imperative not to overlook the potential public health concerns associated with this pathogen, and this study contributes to a better understanding of its mechanisms.

### Supplementary Information

**Additional file 1. Primers used in this study.**

## Data Availability

Not applicable
