# Supplementary material for: Pasteurella multocida infection induces blood–brain barrier disruption by decreasing tight junctions and adherens junctions between neighbored brain microvascular endothelial cells
Source: Vet Res. 2024 Aug 29;55:104. doi: 10.1186/s13567-024-01351-5 (PMC11363436; doi:10.1186/s13567-024-01351-5)
Supplement: Supplementary file 1 — Additional file 1. Primers used in this study. [file 13567_2024_1351_MOESM1_ESM.docx]

**Additional file 1. Primers used in this study.**

| **Targets** | **Sequences (5’-3’)** | **Products (bp)** | **Role** |
| --- | --- | --- | --- |
| HIF-1α | F: CCATGCCCCAGATTCAGGAT | 179 | Detecting the transcription of HIF-1α in HBMECs |
|  | R: TGGGTTCTTTGCTTCTGTGTCT |  |  |
| VEGFA | F: CCCAGTTTTGGGAACACCGA | 153 | Detecting the transcription of VEGFA in HBMECs |
|  | R: CCCCAAAGCACAGCAATGTC |  |  |
| GAPDH | F: GGAGTCCACTGGCGTCTTCA | 240 | Detecting the transcription of GAPDH in HBMECs |
|  | R: GTCATGAGTCCTTCCACGATACC |  |  |
| ZO-1 | F: TGTGGAAGAGGATGAAGATGAAGA | 192 | Detecting the transcription of ZO-1 in HBMECs |
|  | R: GGTGGAAGGATGCTGTTGTC |  |  |
| OCLN | F: TTAACTTCGCCTGTGGAT | 144 | Detecting the transcription of OCLN in HBMECs |
|  | R: TGTGTAGTCTGTCTCATAGTG |  |  |
| CLDN5 | F: CGCCTTCCTGGACCACAACAT | 116 | Detecting the transcription of CLDN5 in HBMECs |
|  | R: CCAGCACCGAGTCGTACACTT |  |  |
| IL-6 | F: ACTCACCTCTTCAGAACGAATTG | 149 | Detecting the transcription of IL-6 in HBMECs |
|  | R: CCATCTTTGGAAGGTTCAGGTTG |  |  |
| TNF-α | F: CGAGTGACAAGCCTGTAG | 165 | Detecting the transcription of TNF-α in HBMECs |
|  | R: GGACCTGGGAGTAGATGA |  |  |
| IL-1β | F: ATGATGGCTTATTACAGTGGCAA | 132 | Detecting the transcription of IL-1β in HBMECs |
|  | R: GTCGGAGATTCGTAGCTGGA |  |  |
| NF-κB | F: GAAGCACGAATGACAGAGGC | 137 | Detecting the transcription of NF-κB in HBMECs |
|  | R: GCTTGGCGGATTAGCTCTTTT |  |  |
| HIF-1α | F: GCUGGAGACACAAUCAUAUTT | | siRNA for suppressing the expression of HIF-1α in HBMECs |
|  | R: AUAUGAUUGUGUCUCCAGCGG | |  |
| NF-κB | F: GUCACUCUAACGUAUGCAAUUTT | | siRNA for suppressing the expression of NF-κB in HBMECs |
|  | R: AAUUGCAUACGUUAGAGUGACTT | |  |
